# Development of a functional human induced pluripotent stem cell-derived nociceptor MEA system as a pain model for analgesic drug testing

**DOI:** 10.3389/fcell.2023.1011145

**Published:** 2023-03-01

**Authors:** Siddharth Nimbalkar, Xiufang Guo, Alisha Colón, Max Jackson, Nesar Akanda, Aakash Patel, Marcella Grillo, James J. Hickman

**Affiliations:** ^1^ Hybrid Systems Lab, University of Central Florida, NanoScience Technology Center, Orlando, FL, United States; ^2^ Hesperos Inc., Orlando, FL, United States

**Keywords:** IPSC, nociceptor, pain model, human, drug testing, MEA

## Abstract

The control of severe or chronic pain has relied heavily on opioids and opioid abuse and addiction have recently become a major global health crisis. Therefore, it is imperative to develop new pain therapeutics which have comparable efficacy for pain suppression but lack of the harmful effects of opioids. Due to the nature of pain, any *in vivo* experiment is undesired even in animals. Recent developments in stem cell technology has enabled the differentiation of nociceptors from human induced pluripotent stem cells. This study sought to establish an *in vitro* functional induced pluripotent stem cells-derived nociceptor culture system integrated with microelectrode arrays for nociceptive drug testing. Nociceptors were differentiated from induced pluripotent stem cells utilizing a modified protocol and a medium was designed to ensure prolonged and stable nociceptor culture. These neurons expressed nociceptor markers as characterized by immunocytochemistry and responded to the exogenous toxin capsaicin and the endogenous neural modulator ATP, as demonstrated with patch clamp electrophysiology. These cells were also integrated with microelectrode arrays for analgesic drug testing to demonstrate their utilization in the preclinical drug screening process. The neural activity was induced by ATP to mimic clinically relevant pathological pain and then the analgesics Lidocaine and the opioid DAMGO were tested individually and both induced immediate silencing of the nociceptive activity. This human-based functional nociceptive system provides a valuable platform for investigating pathological pain and for evaluating effective analgesics in the search of opioid substitutes.

## 1 Introduction

Nociceptors are a subset of sensory neurons specialized for pain sensation. The sensation of pain is a protection mechanism that alerts the body to toxic or harmful condition or insults. However, the unpleasant physical and emotional physiological responses associated with pain response need to be controlled in order to allow for normal physiological activity. Current medications for control of significant and chronic pain relies heavily on opioids, which are effective but can have severe side effects associated with multiple undesired complications, including dependence or addiction (Benyamin et al., 2008). Widespread use of opioids has led to a global crisis of opioid addiction, a severe pathology that causes major lifestyle and socioeconomic consequences (Dasgupta et al., 2018). Therefore, it is imperative to develop systems to evaluate novel drugs that maintain efficacy for pain control but have less undesirable side effects.

Traditional drug and disease testing pipelines rely heavily on animal-based systems, however, due to the lack of translatability, many drugs that make it past animal trials have failed in subsequent human trials due to the species gap between animals and humans (van der Worp et al., 2010; Wong et al., 2019). Additionally, the use of animals in preclinical drug investigations is accompanied with ethical considerations, and can be expensive, however both of these concerns would be alleviated or greatly reduced through the use of human-on-a-chip based models (Rai and Kaushik, 2018). *In vitro* biological models are increasingly gaining attention as viable platforms for disease study and drug development, due to their potential for modeling efficiency, increased reproducibility, and the incorporation of human tissues leading to superior bench to bedside translational results as compared to animal models (Liu et al., 2013). The development of a human cell-based nociceptive system would help facilitate drug development for pain control by providing a high content screening platform applicable for the development of new therapeutics.

The advent of stem cell technology has provided an avenue to allow these types of models to be developed. Modeling human diseases using induced pluripotent stem cells (iPSCs) has become a prominent branch of research due to the pluripotency and easy accessibility of these cells. IPSCs can be derived from fibroblasts or blood cells by de-differentiation, after which they can be used for downstream differentiation into multiple cell types. This would allow for the evaluation of specific genetic variants of diseases in a wide variety of tissue types by either deriving iPSCs from patients expressing a specific genotype, or by using methods such as CRISPR/CAS9 to induce genetic changes. The proliferative potential of iPSCs also confers the possibility for limitless availability of cell supplies.

Differentiation of nociceptors from human stem cells has been previously reported. A representative study indicated the differentiation of nociceptors from hiPSCs by utilizing a combined small molecule inhibition protocol, over the course of about 10 days (Chambers et al., 2012). The generated nociceptors responded to ATP, but only a small population of the cells (1–2%) responded to capsaicin based on calcium imaging. Another study reported the differentiation of nociceptive neurons from human embryonic stem cells (ESCs) by using retinoic acid for caudalization and BMP-4 for dorsalization. This induction process requires about 1 month, and another month was needed for the development of functional nociceptive neurons (Boisvert et al., 2015). The generated nociceptive neurons responded to capsaicin (1 μM, 14% of the neurons by Calcium imaging) and another TRPV1 agonist, α, *ß*-methyleneadenosine-5′-triphosphate lithium (30 μM, 21% of the neurons by Calcium imaging). IPSC-derived nociceptors have been utilized for pain research in multiple studies. For example, by using patient-specific iPSCs, inter-individual differences in pain sensation have been elucidated by the genetic variations in nociceptor ion channels (McDermott et al., 2019; Mis et al., 2019). The study demonstrated an exciting approach that bridges human genetics, physiology, and patient outcomes by using iPSC-derived neurons (Meents et al., 2019; Naka, 2019).

This study aimed to develop a human iPSC-derived nociceptive model integrated with solid state microelectrode array (MEA) technology to facilitate the study of pain and drug development. We characterized the iPSC-derived nociceptors according to established protocols, developed a serum free nociceptor medium that supported the maturation and long-term culture of the human nociceptors, established their functional maturity, and then demonstrated the ability of this pain model to be used as a platform for analgesic drug testing and development.

## 2 Materials and methods

### 2.1 DETA coating of coverslips and MEAs

18 mm glass coverslips, and MEAs were placed on a ceramic rack and plasma cleaned using a Harrick plasma cleaner supplemented with an oxygen tank. A 0.1% v/v trimethoxysilyspropyldiethylenetriamine (DETA) solution was prepared in a N_2_ operated glovebox chamber using distilled toluene as the solvent. The plasma cleaned coverslips and MEAs were then placed in a glass beaker containing the 0.1% DETA solution, which was heated to approximately 90°C–100°C for 30 min on a hot plate. The beaker was then cooled down to room temperature over the course of 30 min. The ceramic racks holding the coverslips were rinsed in three serial toluene baths, placed in another beaker containing only distilled toluene, and heated again for 30 min to a temperature of around 90°C–100°C. The surfaces were then removed from the distilled toluene and left overnight in an oven at 110°C. Once the surfaces had been cured, they were characterized *via* X-ray photoelectron spectroscopy (XPS) analysis to verify the N/Si ratio, the key characteristic of this surface modification in terms of promoting cellular attachment (Wilson et al., 2011). The DETA coated coverslips and MEAs are then modified with a coating of polyornithine 15 μg/mL and Laminin 1 μg/mL + Fibronection 10 μg/mL.

### 2.2 Differentiation of nociceptors from human iPSC cell lines

The iPSC cell line ND41865 (from Coriell Institute) was reprogrammed from fibroblasts derived from a healthy male subject. The integrity of the chromosomes and the pluripotency has been well characterized. This iPSC line has successfully been differentiated into a multitude of other cell types in our lab, therefore it was chosen for this proof of principle study. Cells of this iPSC line were cultured in mTESR1 medium (StemCell Technologies, 85,850) on Matrigel (Corning, 354,230) coated six well tissue culture plates. 1 µG Matrigel coating solution was prepared by adding 100 µL of Matrigel to 6 mL of DMEM/F12 medium (FisherScientific, 21041-025), which was adsorbed to a six well plate. ROCK inhibitor (StemCell Technologies, 72,304) Y-27632 was added to mTESR1 at a concentration of 5 µM for the first 20 h of culture after thawing or passaging. Full medium changes were done daily and cells were passaged to another Matrigel coated 6-well plate when they reached 70% confluency for downstream differentiation.

To drive the differentiation of the iPSC cell line to a nociceptor phenotype, LSB combined with three small molecule inhibitors was used (Chambers et al., 2012). Specifically, once the replated iPSC cell lines reached 70% confluency, differentiation was initiated by switching the original mTESR1 medium to Knockout Serum Replacement (KSR) medium supplemented with the signaling factors LDN 193189 (Tocris), SB 431542 (Tocris), SU 5402 (Tocris), CHIR 99021(Tocris), and DAPT (Tocris). KSR medium was prepared by using knockout DMEM as a base medium supplemented with KSR, L -glutamine (200 mM), 10 mM MEM non-essential amino acids, and *ß* mercaptoethanol (55 mM). As differentiation progressed the KSR medium was gradually replaced by N2B medium while the signaling factor concentrations were maintained. N2B medium is equivalent to DMEM/F12 supplemented with 1X N2 (Life Technologies, 17,502-048) and 20 μg/mL Insulin (Serologicals Corp 2002712 or equivalent). The ratio of KSR medium to N2B medium progressed as follows; Day 0–3: 100% KSR; Day 4–5: 75%KSR, 25% N2B; Day 6–7 50% KSR 50% N2B; Day 8–9 25% KSR 75% N2B; Day 10–11 100% N2B. At day 12, the cells were fed with maintenance medium which was made using the N2B medium listed, supplemented with BDNF (25 ng/mL) (Cell Sciences), GDNF (25 ng/mL) (Cell Sciences) and human *ß*-NGF (25 ng/mL) (R&D Systems). In order to avoid an osmotic shock, the maintenance medium was gradually changed from the N2B based maintenance medium to a 1:1 mixture of neurobasal and N2B for 2 days, and finally on day 4 the cells were switched to the neurobasal based nociceptor medium, reducing the osmolarity from 330 mOSM/kg to 230 mOSM/kg. The final medium to be used was a Neurobasal-based medium supplemented with B27 100X (1%), Glutamax (1%), BDNF (25 ng/mL), GDNF (25 ng/mL), NT3 (25 ng/mL), IGF (10 ng/mL), and human *ß*-NGF (25 ng/mL). The full medium switch schedule is listed in Supplementary Table S1, and the product information for the small molecules can be found in Supplementary Table S2.

### 2.3 Immunocytochemistry

To confirm the phenotype of the differentiated neural cells, immunocytochemistry was performed using a variety of nociceptive-specific markers. The cells were fixed with 4% paraformaldehyde (PFA) solution for 15 min, and rinsed 3 times with 1X phosphate buffered saline (PBS) for 5, 10, and 15 min. The cells were then permeabilized with 0.1% Triton for 15 min, and incubated in a blocking solution (5% Donkey serum, 0.5% BSA in PBS) for 1 h at room temperature. Next the cells were incubated in blocking buffer containing the primary antibodies, at 4°C overnight. The following day, the cells were washed with PBS for 5, 10, and 15 min, after which secondary antibodies diluted in blocking buffer were added and incubated at room temperature for 2 h protected from light. The cells were rinsed with PBS for 5, 10, and 15 min with 4′, 6-diamidino-2-phenylindole [DAPI solution] (1:1000) added to the PBS during the 10 min wash. The coverslips were then mounted on a glass slide using ProLong^TM^ Gold antifade mountant from ThermoFisher and imaged using a Zeiss Axioskop two mot plus spinning disk confocal microscope. Primary antibodies are listed in Table 1. The secondary antibodies include Goat-anti-Mouse-568 (Invitrogen, 1:250), Goat-anti-Mouse-488 (Invitrogen, 1:250), Goat-anti-Rabbit-488 (Invitrogen, 1:250), Goat-anti-Rabbit-568 (Invitrogen, 1:250), Goat-anti-chicken-647 (Invitrogen, 1:250), Goat-anti-rat-488 (Invitrogen 1:250), Donkey-anti-Rabbit-488 (Invitrogen, 1:250), and Donkey-anti-Goat-568 (Invitrogen, 1:250).

**TABLE 1:** The list of primary antibodies utilized in this study.

Antibody	Species	Company	Catalog no	Dilution factor
TrpV1	Rabbit	Invitrogen	PA1-748	1:200
TrKA	Mouse	EMD Millipore	MABN681	1:100
NF	Chicken	EMD Millipore	AB5539	1:1,000
P2X3	Rabbit	Neuromics	RA10109	1:200
P2X4	Goat	Invitrogen	PA5-37880	1:50
Peripherin	Mouse	Santa cruz biotech	Sc-377093	1:200
Substance P	Rat	EMD Millipore	MAB356	1:100
cGRP	Mouse	Abcam	Ab81887	1:100
MOR	Rabbit	Abcam	Ab10275	1:800

### 2.4 Whole-cell patch clamp electrophysiology

Current-clamp and voltage-clamp recordings were performed using a Zeiss, upright microscope (Axioscope, FS2, Carl Zeiss, Germany) equipped with a multiclamp 700B amplifier. Borosilicate glass patch pipettes (BF 150–86–10; Sutter Instrument Company), with a resistance of 6–10 MΩ, were made using a Sutter P97 pipette puller (Sutter Instrument Company). The pipette (intracellular) solution contained 140 mM K-gluconate, 4 mM NaCl, 0.5 mM CaCl_2_, 1 mM MgCl_2_, 1 mM EGTA, 5 mM Na_2_ATP, 5 mM HEPES base, and 5 mM HEPES acid. The pH and osmolarity were adjusted to 7.2 and 280 mOsmole, respectively. The nociceptor maturation medium was used as the extracellular solution for all patch-clamp experiments. Following the formation of a Giga-Ω seal and membrane puncture, the cell capacitance was compensated. Signals were filtered at 3 kHz and digitized at 20 kHz using a Digidata 1322A interface (Axon Instruments). Data recording and analysis were performed using the pClamp10 software (Axon Instruments). Membrane potentials were corrected by subtraction of a 15 mV tip potential, which is the liquid junction potential between intracellular solution and extracellular solution, and was calculated using Axon’s pClamp10 program.

Sodium and potassium currents were measured using a voltage-clamp protocol of 15 pulses from −20 to +120 mV with a stepwise incremental of 10 mV and 120 ms duration, while the holding voltage was −70 mV as detailed previously (Akanda et al., 2009). Whole-cell capacitance and series resistance were compensated and a p/6 protocol was used. The access resistance was less than 20 MΩ. Induced single and repetitive action potentials (APs) were recorded in current-clamp mode using 1-s depolarizing current injections with a stepwise incremental of 10 pA from a −70 mV holding potential. Other parameters in the protocol were optimized during the recording due to cell to cell variations. Spontaneous activity was recorded in gap-free mode. The data were analyzed using pClamp 10 software (Axon Instrument, Foster City, CA, United States) and quantified using Microsoft Excel. More than 10 cells were analyzed for the experiments testing the response to each chemical, and more than 20 cells recorded in total.

### 2.5 MEA analysis

Cells were cultured on custom designed microelectrode array (MEA) chips coated with DETA as outlined above followed by an ECM coating; Poly-L-Ornithine (Sigma Aldrich) at 15 μg/mL overnight at room temperature followed by laminin (Fisher Scientific) at 1 μg/mL and fibronectin (Sigma Aldrich) 10 μg/mL for overnight at room temperature. Cells were plated at a density of 700 cells/mm^2^ and maintained for a minimum 14 days before neuronal activity was recorded using an INTAN-based extracellular recording system. Baseline/spontaneous cellular activity was recorded for 5 min, followed by cell response to ATP doses at 500 nM, 50 μM, and 5 mM, each recorded for a period of 3 min. For experiments with Lidocaine or opioid, a dose of 100 μL lidocaine (10 mM) or DAMGO (50 μM) was added to the 1 mL medium in the recording chamber, and activity was recorded for a period of 3 min. Remaining background noise was subtracted from previous recordings. At the end of the recordings, the systems were rinsed with maintenance medium to remove any residual ATP, lidocaine or DAMGO, and replenished with fresh medium. The evoked response or recorded cell activity was then analyzed using Anaconda with Python software. The standard deviation was set to ± 5 and the signals were then passed through a 100 Hz high pass filter and 60 Hz notch filter. 0.1 Hz was considered to be the cutoff frequency and any channel with a frequency above that was considered to be a real biological electrical signal. For each data set, at least three batches of experiment were analyzed, with one or more biological replicates for each batch. One-way ANOVA were utilized to compare the statistical difference between samples with a particular treatment and the untreated baseline control.

## 3 Results

### 3.1 Differentiation nociceptors from iPSCs

**FIGURE 1:**
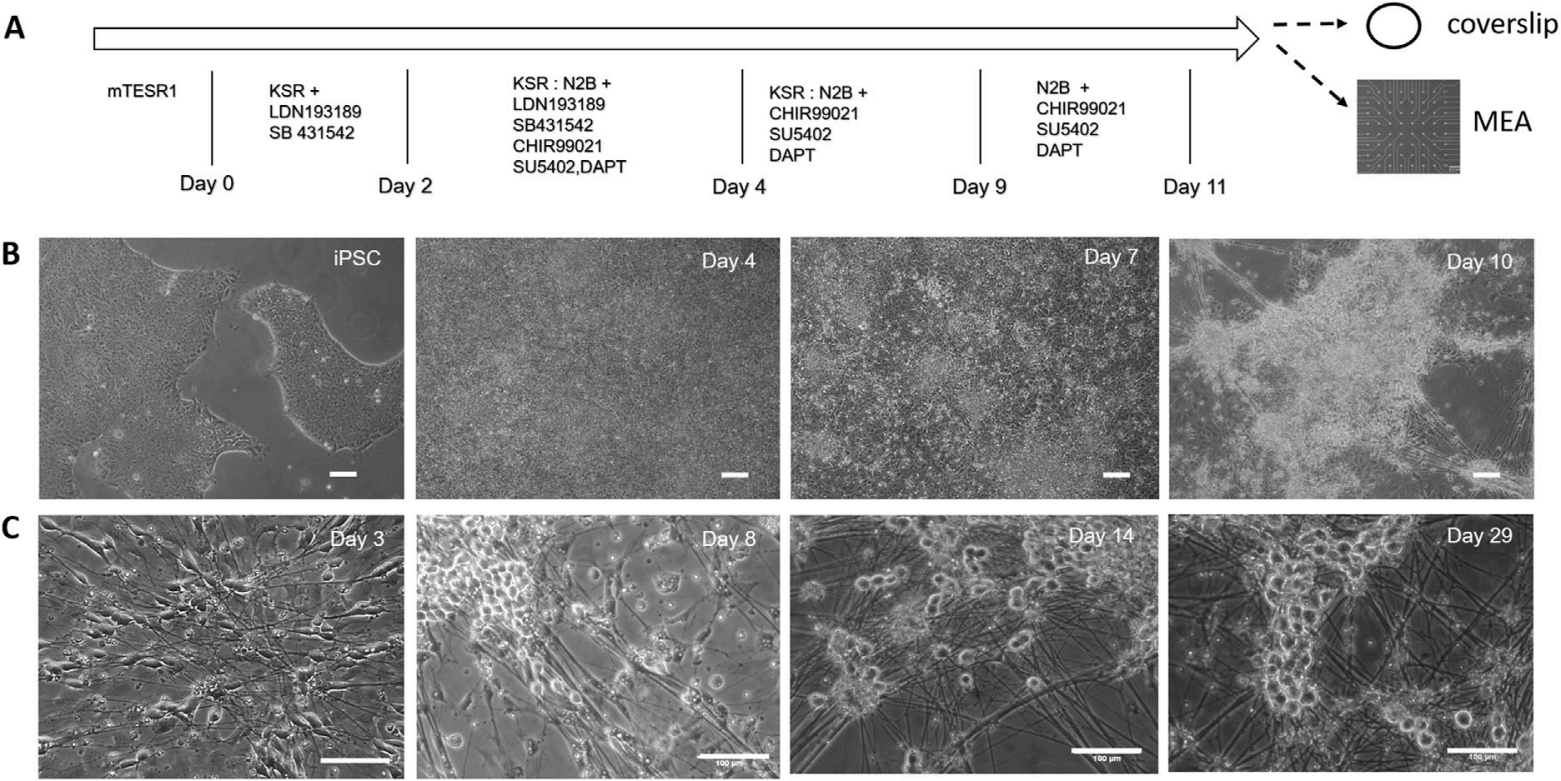
Differentiation of nociceptors from hiPSCs characterized by phase microscopy. **(A)** Outline of the iPSC-nociceptor differentiation process and cell culture procedure. Timeline showing medium changes and inclusion of small molecules at different days during the differentiation process. At the completion of the differentiation process, the cultures were harvested using trypsin and replated onto coverslips for characterization and MEAs for functional analysis. **(B)** Phase images showing the morphological progression of the iPSC-nociceptor culture during the differentiation process. Once the iPSC colonies reached 70% confluency, differentiation is induced. As the differentiation progressed, the confluent colonies started to form clusters, which became prominent at Day 10 of differentiation and the nociceptors and differentiation may already have been initiated as suggested by the axonal processes arising from the clusters. Scale bar: 100 μm. **(C)** Phase images indicate the morphological progression of the iPSC-nociceptor culture after replating during cell maturation. During the initial phase the cells started showing spindle shaped bipolar morphology. After a few days some of the processes started moving towards each other tending to form the pseudo unipolar morphology. Post 2 weeks the soma tended to form clusters similar to clusters of sensory neurons found in DRGs. Scale bar: 100 μm.

Nociceptors were differentiated from iPSCs according to a modified LSB3i protocol initially developed by Chambers et al. (2012). Neuralization was induced *via* dual SMAD inhibition with SB431542 and LDN-193189 (LSB) (Chambers et al., 2009). This was followed by treatment with three small molecule inhibitors, CHIR 99021, SU5402 and DAPT (3i), to drive the differentiation down the neural crest lineage, and accelerate generation of postmitotic peripheral neurons. The diagram of the differentiation process and cell culture procedure is shown in Figure 1A. The nociceptive identity of the differentiated neurons was determined by examining expression of nociceptor specific markers including vanilloid receptors and their electrophysiological profile. The morphological progression of the culture during the differentiation stage is shown in Figure 1B. At the end of the differentiation process, most neurons formed clusters with axonal bundles projecting from the clusters. If kept in the same medium as in D11 (Chambers et al., 2012), the clustering process continued and the majority of the cultures generally detached from the surface after about a week in culture, which makes it difficult for the analysis and application of these cells.

In order to promote a robust nociceptive culture, a unique defined, serum free medium that supports maturation and long term culture of the differentiated nociceptors was developed (Supplementary Table S3). The nociceptor culture was able to be maintained for at least 28 days *in vitro* with healthy morphological and electrophysiological function. To evaluate the morphology of the iPSC-derived nociceptors, the cells were replated onto DETA coverslips after differentiation and monitored under phase contrast microscopy (Figure 1C). At day 2 during the initial stages of growth, the cells began to exhibit a spindle shaped bipolar morphology, with axons growing in a polar orientation. After 7 days *in vitro*, the cells initiated conversion from bipolar to pseudo-unipolar morphology, which is representative of the *in vivo* morphology of nociceptors. Additionally, at this time point, the cells began to form clusters exhibiting morphology similar to what is found in human dorsal root ganglion (DRG), structures where nociceptors are located *in vivo*. Both the morphological and functional analyses were conducted prior to day 28 after cell replating.

### 3.2 Characterization of iPSC-nociceptors by immunocytochemistry

**FIGURE 2:**
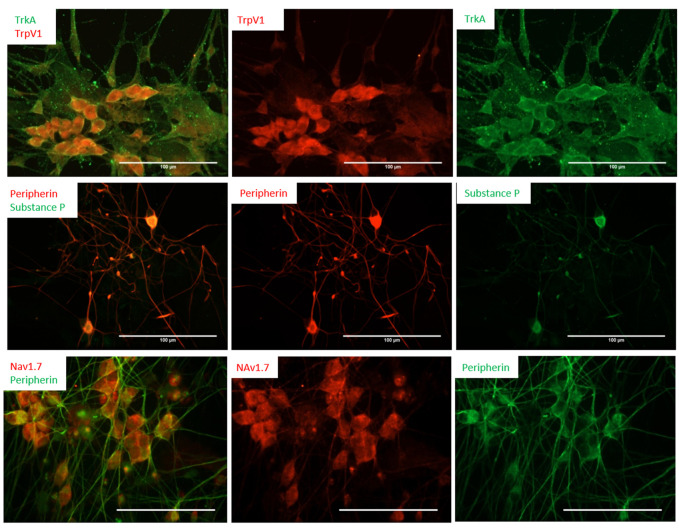
Differentiation of nociceptors from human iPSCs was characterized by immunocytochemistry. The cells were immunostained for the expression of NGF and the VR1 receptors TrkA and TrpV1 (Day 14, top panel), as well as the neuropeptides Substance P (Day 16, middle panel) and Nav1.7 (D21, bottom panel). Scale bar: 100 μm.

The iPSC-derived neurons were characterized by immunocytochemistry (ICC) to confirm the nociceptive phenotype (Figure 2). The differentiated cells were first immunostained for the expression of Tropomyosin receptor kinase A (TrkA), and transient receptor potential cation channel subfamily V member 1 (TrpV1). TrkA is a receptor for Nerve Growth Factor (NGF), a neurotrophic factor that has been found to play a pivotal role in the differentiation and function of the nociceptive system (Barker et al., 2020), and has been included in the maintenance medium. TrpV1s, also known as the capsaicin receptor and the vanillioid receptor1 (VR1), are ionic channels that respond to noxious stimuli such as heat, low pH and capsaicin, which leads to an influx of calcium ions triggering a series of mechanisms resulting in the detection of pain. The expression of these two nociceptor markers (TRPV1, TRKA), combined with the general sensory neuron marker Peripherin, were examined at multiple days (days 14, 23, and 30) post-differentiation. The results consistently indicated almost all the neurons were positive to these two nociceptor markers. Similar results were obtained from the D21 staining for Nav1.7*.* In addition to these ion channels, the expression of Substance P (Sub P), a neuropeptide associated with the pain pathway, was also evaluated. Sub P is a characteristic neurotransmitter found within a subpopulation of nociceptive sensory neurons which modulate nociception, or pain sensation, through interaction with its receptors (neurokinin) and subsequent downstream signaling pathways (Chang et al., 2019). Sub P was found to be expressed in a subset of these iPSC-nociceptors, in agreement with literature reports (Stucky et al., 2007). Moreover, all the differentiated neurons were positive for Nav1.7, a sodium channel marker expressed at high levels specifically on nociceptors and sympathetic ganglion neurons. This channel is considered the “volume knob” to establish the gain of nociceptive signaling (Kingwell, 2019), and has been found to play a significant role in pathological pain (Hameed, 2019).

**FIGURE 3:**
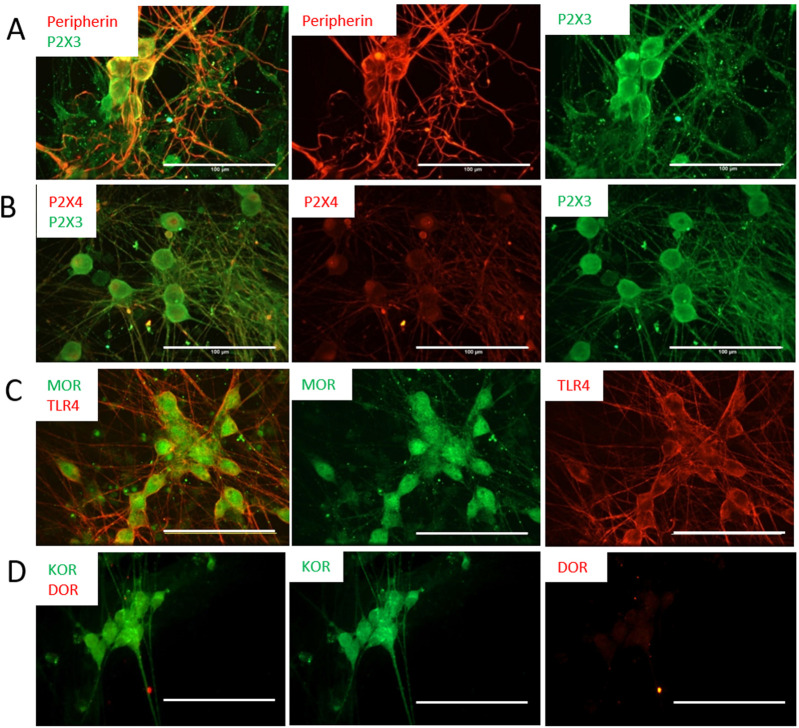
Expression of ATP and opioid receptors by the nociceptors was characterized by immunocytochemistry at D21. The cells are positive for the P2X3 antibody **(A)**, but negative to the P2X4 antibody **(B)**, confirming their nociceptive identity. **(C)** The cells showed positive staining to the µ-opioid (MOR) and TLR4 receptors. **(D)** The cells indicated positive staining to KOR but were negative for DOR. Scale bar: 100 μm.

Extracellular ATP has been shown to be involved in the pain sensation mechanism through P2X3 gated receptors on peripheral sensory neurons. P2X3 channels are found in C- and Aδ-primary afferent neurons in most tissues, and are highly specific to pain detection, and significantly contribute to pain sensitization (Fabbretti, 2013). The presence of these receptors, but not another type of ATP receptor, P2X4, was confirmed by ICC at day 14 (Figure 3). It is well-known that nociceptors are targets for opioids during pain treatment, so in order to determine the validity of applying these cells for opioid-related studies, the expression of different types of opioid receptors was examined. As demonstrated in Figure 3, these cells were positive for the *µ*- and *κ*-opioid receptors (MOR and KOR, respectively), but negative for the *δ*-opioid receptor (DOR). *In vivo*, the activity of nociceptors is also responsive to immune-related factors, such as Lipopolysaccharides (LPS), a major component of the outer membrane of Gram-negative bacteria and then toll-like receptor 4 (TLR4) mediates the response triggered by LPS (Park and Lee, 2013). To evaluate these nociceptor’s potential to examine the comorbidity of pain with immune response, the cells were also analyzed for the expression of TLR4, which confirmed the expression of this important receptor.

### 3.3 Functional characterization of the iPSC-nociceptors by patch clamp electrophysiology

**FIGURE 4:**
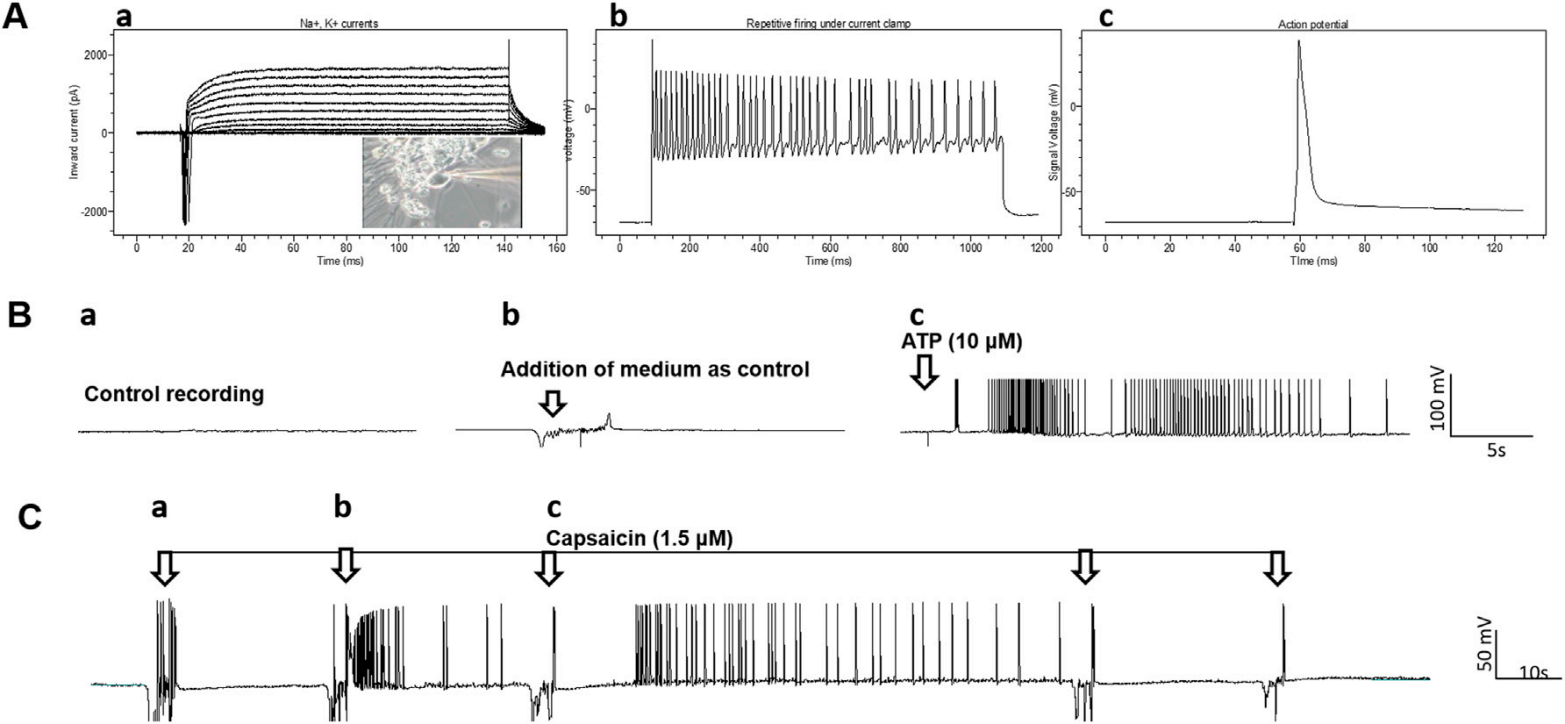
Patch clamp analysis of the iPSC-derived nociceptors. **(A)**, **a)** Recording of Na^+^, K^+^ currents under voltage clamp conditions. The inset is the phase image of the cell patched, **b)** recordings of repetitive firing under current clamp conditions and **c)** action potential. **(B)** Response of iPSC-nociceptors (Day 16) to ATP recorded in patch clamp gap free mode. **a)** Baseline recording indicated no spontaneous firing, **b)** addition of medium didn’t induce neuronal activity except random artifacts and **c)** addition of ATP (10 µM) induced robust firing after a short delay. **(C)** Response of iPSC-nociceptors to Capsaicin recorded by patch clamp gap free mode. Initial addition of Capsaicin didn’t induce neuronal activity except random artifacts, while additional addition of capsaicin (1.5 µM) induced robust firing after a short delay. However, further administration induced no additional activity, which is reminiscent of the therapeutic effects of Capsaicin for pain induction.

The electrophysiological properties of the neurons were analyzed by patch clamp recordings; resting membrane potential, Na^+^ currents, K^+^ currents, repetitive action potential (AP) firing, and spontaneous AP firing (Figure 4). Whole cell patch clamp was used to determine the electrical activity and response of the nociceptors to the nociceptive modulator ATP and to the noxious stimuli capsaicin (Figures 4B, C). Spontaneous firing recorded under gap free conditions was initially recorded, and as a vehicle control to ensure no activity was induced by medium addition, the same volume of medium used when adding the testing compounds was also added to the cells, which only triggered sporadic artifacts. Afterwards, addition of 30 μL of a 1 mM solution of ATP to the recording chamber (final concentration 10 μM) induced a burst of APs (Figure 4B) in most of the neurons recorded (10 out of 13 neurons recorded). Similarly, the response of these nociceptors to capsaicin was tested and a small number of nociceptors (2 out of 16 neurons recorded) were responsive to capsaicin (Figure 4C). This is similar to a previous report from iPSC-nociceptor studies that only a small percentage (1–2%) of nociceptors respond to capsaicin (Chambers et al., 2012; Boisvert et al., 2015). Both functional properties confirmed the identity and electrophysiological properties of these iPSC-nociceptors.

### 3.4 Modeling the ATP-induced nociceptive response and evaluation of an anesthetic drug utilizing a solid state MEA system

**FIGURE 5:**
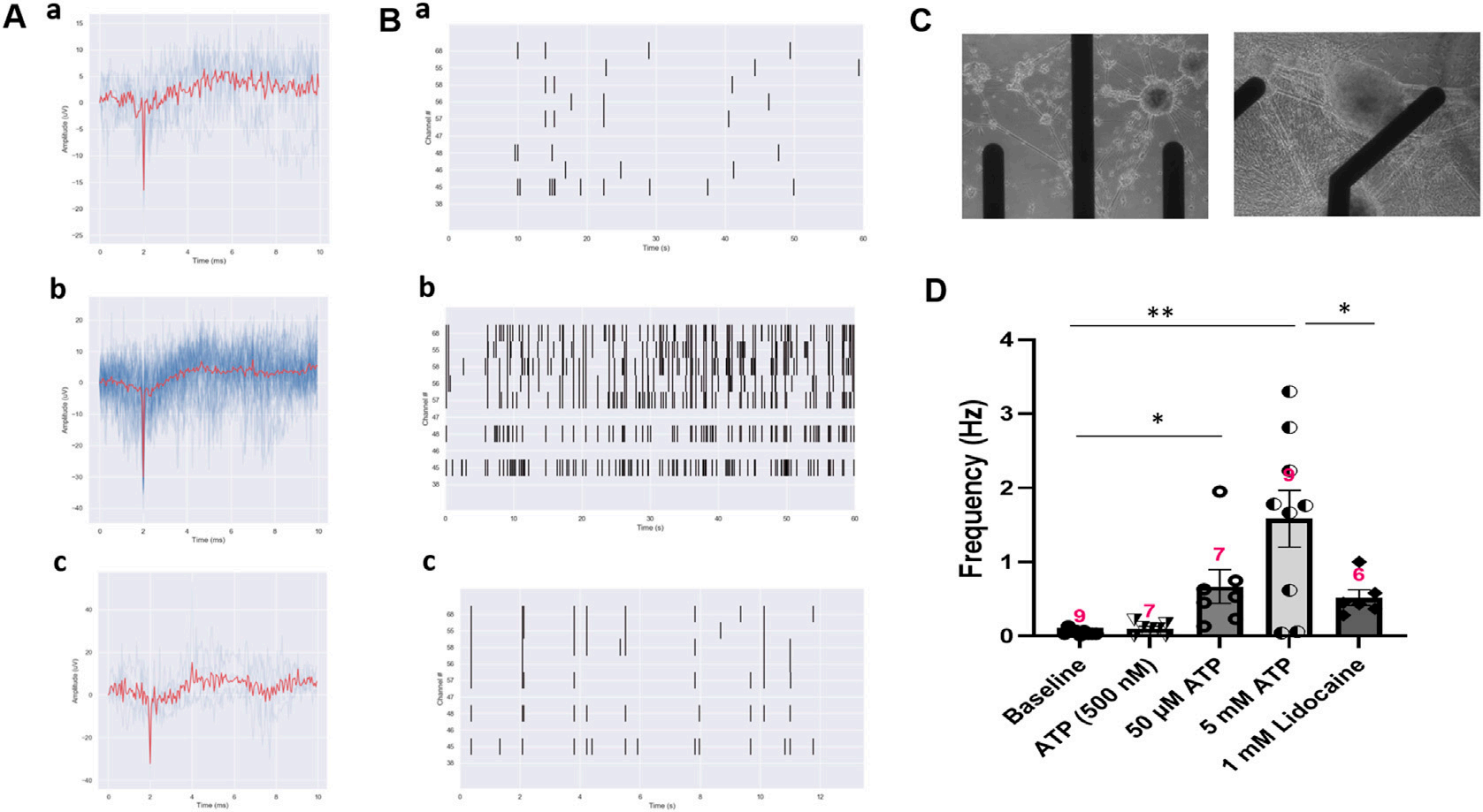
Activity of iPSC-nociceptors recorded on MEAs. **(A)** Representative MEA action potential responses of iPSC-nociceptors demonstrating the excitation effect of ATP. **a)** baseline activity, **b)** activity after addition of ATP (5 mM) and **c)** activity after the subsequent addition of lidocaine (1 mM). **(B)** Raster plot demonstrating the change of neural activity as described in **(A)**. **(C)** Phase images of the electrode with iPSC nociceptors plated on an MEA. **(D)** Graph of the neural firing frequency as described in **(A)**. Data presented are Mean + Standard Error. One-way ANOVA, * <0.05; ** <0.005. Number of biological replicates from at least three batches of experiments were indicated in each data bar in the graph.

While single cell patch clamp electrophysiology measures the response of a single neuron, the populational response of nociceptors is better evaluated through the use of microelectrode array (MEA) recordings (Figure 5). The iPSC-derived nociceptors were cultured on MEAs for 14 days before testing. The majority of the iPSC-nociceptors had low spontaneous activities at rest based on observations from the patch clamp experiments. Similarly, the baseline activity level of these neurons on MEAs was also low. This is reasonable since the nociceptor activity is typically quiet under homeostatic conditions and environments. However, similar to what was seen in the patch clamp results, the addition of ATP induced an increase in firing frequency as compared to baseline recordings, and the firing frequency increased as the dosage of ATP increased (Figure 5D). Addition of Lidocaine as a functional control was observed to eliminate the majority of these APs. The decrease in the firing frequency by the addition of lidocaine not only demonstrated that the APs seen were induced as a biological response to ATP, but also simulated the analgesic effects of a drug in this iPSC-nociceptor *in vitro* system. It was also observed in Figure 5D that the neural activity after Lidocaine dosing was still a bit higher than baseline. This is mainly because the dosage of Lidocaine was not high enough in some cases when the populational activity was very strong, or the drug diffusion rate in the recording chamber was not high which delayed the onset of the overall silencing effect.

### 3.5 Response of the iPSC-derived nociceptors to an opioid

**FIGURE 6:**
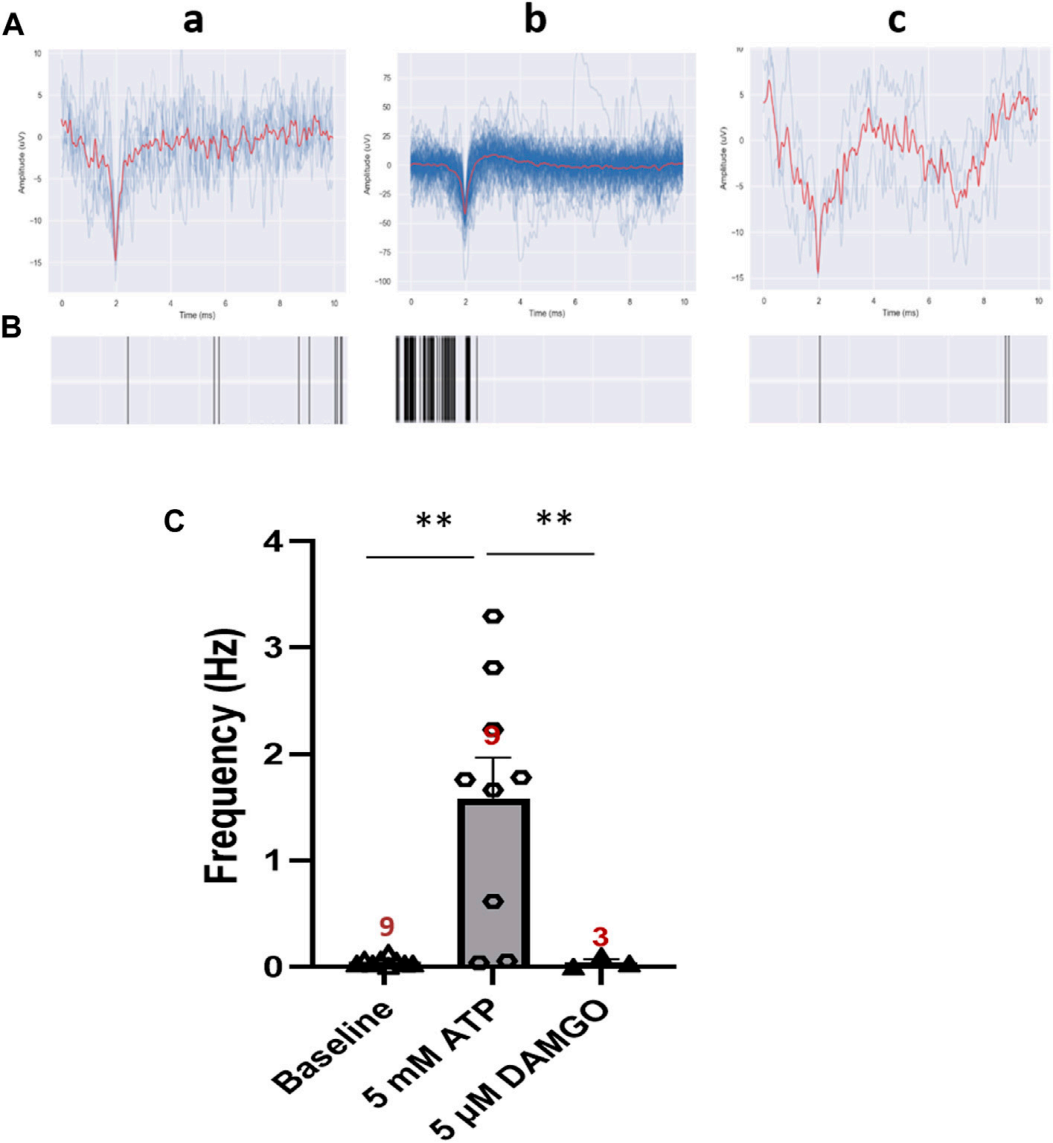
Inhibition effect of DAMGO on ATP-induced neural activity of iPSC-derived nociceptors recorded on MEAs. **(A)** Representative MEA action potential responses of iPSC-derived nociceptors demonstrating the inhibition of ATP-induced activity by DAMGO (5 μM) **a)** baseline activity, **b)** activity after addition of ATP (5 mM) and **c)** activity after the subsequent addition of DAMGO (5 μM). **(B)** Raster plot demonstrating the change of neural activity as described in **(A)**. **(C)** Graph of the neural activity change as described in **(A)**. Data presented are Mean + Standard Error. One-way ANOVA, * <0.05; ** <0.005). Number of biological replicates from at least three batches of experiments were indicated in each data bar in the graph.

Opioids are still the most effective and commonly prescribed drugs for severe pain and generally prescribed to patients dealing with serious injury, pain after surgery, cancer, or chronic pain. In order to validate this iPSC-nociceptor based pain model as a platform for the investigation of opioids and opioid alternatives, it was essential to confirm their responsiveness to opioids. DAMGO [(D-Ala2, N-MePhe4, Gly-ol)-enkephalin], a synthetic opioid peptide with µ-opioid receptor affinity, was utilized as a test therapeutic. In this experiment, ATP was used to stimulate iPSC-nociceptors on MEAs to simulate pain. The neural activity was recorded before and after each addition of ATP at 5 min intervals. A dose of 100 µL of 50 µM DAMGO was added to the MEA chamber after the conclusion of the ATP dosing (final concentration 5 µM), and the activity was recorded for another 5 min. As shown in Figure 6, the activity of these neurons was increased significantly upon ATP addition, then reduced to baseline level by the application of DAMGO, reproducing the expected effect of an opioid for pain suppression in this solid state model system.

## 4 Discussion

In this report, functional nociceptive neurons were differentiated from human iPSCs, and a novel serum free, defined, nociceptor medium was developed that is able to support the cells for at least a month while maintaining good morphology and function. The nociceptive phenotype was characterized by immunocytochemistry and function by patch clamp electrophysiology. These cells were then integrated onto a solid-state MEA chip system to monitor their response to ATP in a dose dependent manner and for the evaluation of the effect of analgesic drugs.

The iPSC-derived nociceptors expressed TrpV1, Na1.7, and P2X3, the major molecular markers associated with nociceptive function and pathological pain, and functional tests further confirmed the response of the cells to relevant stimuli in this high content screening system. TRPV1 is an important integrator of responses to inflammatory mediators. Sensitization of TRPV1 during chronic pain is believed to contribute to the transduction of noxious signaling for normally innocuous stimuli, making the search for novel therapeutics targeting TRPV1 an active area of research (Immke and Gavva, 2006). ATP is a co-transmitter in neurons from both the peripheral and central nervous system and ATP receptors are widely expressed on non-neuronal cells as well as neurons. Fine control of ATP and specific ATP receptor operation are crucial elements of the crosstalk between neuronal and non-neuronal cells (Burnstock and Sawynok, 2010). ATP can be released from damaged or dying cells, or healthy cells as a physiological signaling mechanism. Noticeably, ATP is released from many non-neuronal cell types during mechanical deformation in response to shear stress, stretch or osmotic swelling, as well as hypoxia and stimulation by various agents. Internal ATP is released from most cells in response to inflammation, injury, stress and distension (Bodin and Burnstock, 2001). Currently for purine and pyrimidine receptors, seven P2X ionotropic receptor subtypes and eight P2Y metabotropic receptor subtypes are recognized. P2X3 and P2X2/3 hetero-multimer subtypes have been found localized mainly on nociceptive sensory neurons in dorsal root ganglia (Chen et al., 1995), and accumulative evidence indicates that P2X3 receptors are involved in initiating pain and for neuronal sensitization especially that involved in neuropathic pain (Chizh and Illes, 2001; Hilliges et al., 2002) (Fabbretti, 2013). P2X3 is also pre-synaptically expressed at central spinal terminals of afferent neurons, where ATP further sensitizes pain signals en route to the brain. Therefore, internal ATP and its receptor P2X3 represents an important mechanism for pathological pain, especially neuropathic pain (Inoue et al., 2005). Selective drugs that can inhibit ATP-induced nociceptor activity may lead to therapies that block inappropriate chronic signals at their source (Ford, 2012; Ford and Undem, 2013; Ford et al., 2015).

These nociceptors also expressed TLR4, where TLR4 recognizes exogenous pathogen-associated molecular patterns (PAMPs) and endogenous danger-associated molecular patterns (DAMPs) that can initiate the innate immune response. A study in mice indicated that TLR4 expression in nociceptors mediates the development of nerve-injury induced mechanical hypersensitivity in female mice (Szabo-Pardi et al., 2021). The crosstalk between TLR4 and opioid receptor pathways and their impact on opioid analgesia and immune function has been reported (Zhang et al., 2020). Our ICC analysis demonstrated the expression of MOR and the Kappa opioid receptor (KOR) in the differentiated nociceptors, but not the Delta opioid receptor (DOR). The expression of MOR in nociceptors has been well documented in mice. MOR is found to play essential roles in endogenous nociceptive responses and morphine-induced analgesia (Sora et al., 1997). It is also associated with tolerance and opioid-induced hyperalgesia (Corder et al., 2017). KOR has been reported to be expressed by a transcriptionally distinct subset of peptidergic DRG neurons expressing CGRP, substance P and/or TRPV1, and KOR signaling inhibits nociception, nociceptor sensitization and neurogenic inflammation (Snyder et al., 2018). Although DOR is also considered a potential target for pain treatment, its expression in nociceptive DRG neurons remains controversial (Quirion et al., 2020). Based on studies mostly from rodent models, some studies promote the idea supporting DOR expression in DRG neurons including nociceptors and the co-expression of DOR and MOP in some neurons (Bardoni et al., 2014), while the others infer that DOR is found mainly in large myelinated DRG neurons with a low level of co-expression with MOR (Wang and Wessendorf, 2001). Our results for DOR supports the latter, at least for the nociceptor populations differentiated utilizing the described protocol. To the best of our knowledge, this is the first report of opioid receptor expression in human nociceptors, and further investigation of the signaling through these receptors would shed light on the mechanisms of the various effect of opioids on human nociception.

The ICC analysis suggests the potential utility of these iPSC-nociceptors in a wide spectrum of applications from acute or chronic pain control, inflammation, and opioid effects such as analgesia and tolerance. Integration of the iPSC-nociceptors onto MEA systems has proved its competency for evaluating the effects of the analgesic Lidocaine and the opioid DAMGO. To apply this MEA-nociceptor platform for the testing of capsaicin-induced pain or anesthesia, further investigation is needed to established a particular protocol for obtaining a defined effect, since patch clamp analysis displayed capsaicin elicited action potentials in only a small number of neurons, and both excitation and afterwards inhibition were observed during recording. Compared to single cell patch clamp analysis, the MEA system allows evaluation of a populational neuronal response, hence would facilitate the adaptation of these neurons for circuit integration and neural pathway investigations. While this study utilizes a nociceptor monoculture for pain modeling, it doesn’t include the complex circuitry involved in pain sensation and processing such as glutamatergic and interneurons of the spinal cord dorsal horn, and other central nervous system neurons, such as those found in the PAG and brain stem. However, through the use of MEAs, and additional techniques such as surface patterning to direct neurite growth, this system could be adapted to include additional pain circuit components. These findings indicate that these iPSC-nociceptors where the phenotype was established by the expression of critical markers and electrophysiological functional analysis, can be integrated with an MEA platform, which provides a valuable model for studying pathological pain, comorbidity with the immune response related inflammation, as well as for investigating effective analgesics, especially opiates and their alternatives.

## Data Availability

The raw data supporting the conclusion of this article will be made available by the authors, without undue reservation.
