# Supplementary material for: Development of a functional human induced pluripotent stem cell-derived nociceptor MEA system as a pain model for analgesic drug testing
Source: Front Cell Dev Biol. 2023 Mar 1;11:1011145. doi: 10.3389/fcell.2023.1011145 (PMC10014464; doi:10.3389/fcell.2023.1011145)
Supplement: Supplementary file 1 [file DataSheet1.PDF]

Supplementary Material

1 Supplementary Tables

Days in Differentiation	KSR	N2B	Factors
0	4	0	LDN193189, SB431542
1	4	0	LDN193189, SB431542
2	4	0	LDN193189, SB431542, CHIR99021, SU5402, DAPT
3	4	0	LDN193189, SB431542, CHIR99021, SU5402, DAPT
4	3	1	LDN193189, SB431542, CHIR99021, SU5402, DAPT
5	3	1	CHIR99021, SU5402, DAPT
6	2	2	CHIR99021, SU5402, DAPT
7	2	2	CHIR99021, SU5402, DAPT
8	1	3	CHIR99021, SU5402, DAPT
9	1	3	CHIR99021, SU5402, DAPT
10	0	4	CHIR99021, SU5402, DAPT
11	0	4	CHIR99021, SU5402, DAPT

Supplementary Table 1. Media replacing schedule during iPSC-nociceptor differentiation.

Chemicals	Company	Cat#	Working Concentration
LDN193189	Tocris	6053/10	100 nM
SB431542	Tocris	1614/1	6 µM
CHIR99021	Tocris	4423/10	3 µM
SU5402	Tocris	3300	6 µM
DAPT	Tocris	2634/10	6 µM

Supplementary Table 2. Information of signaling factors in the nociceptor-induction medium.

	Working Concentration	Company	Cat. No
Neurobasal		ThermoFisher	2110349
B27	2%	ThermoFisher	17504001
N2	1%	ThermoFisher	17504028
Glutamax	1%	ThermoFisher	35050079
BDNF	25 ng/ml	Cell Sci	CRB600D
NT3	25 ng/ml	Cell Sci	CRN500D
NGF	25 ng/ml	R&D systems	256-GF
GDNF	25 ng/ml	Cell Sci	CRG400E

Supplementary Table 3. Components of iPSC-nociceptor maturation medium.
